# The use of GPS data loggers to describe the impact of spatio-temporal movement patterns on malaria control in a high-transmission area of northern Zambia

**DOI:** 10.1186/s12942-019-0183-y

**Published:** 2019-08-19

**Authors:** Marisa Hast, Kelly M. Searle, Mike Chaponda, James Lupiya, Jailos Lubinda, Jay Sikalima, Tamaki Kobayashi, Timothy Shields, Modest Mulenga, Justin Lessler, William J. Moss, for the Southern and Central Africa International Centers of Excellence for Malaria Research

**Affiliations:** 1 Johns Hopkins Bloomberg School of Public Health, Baltimore, MD USA; 2 University of Minnesota, School of Public Health, Minneapolis, MN USA; 3 The Tropical Diseases Research Centre, Ndola, Zambia; 4 Macha Research Trust, Choma District, Choma, Zambia; 5 Ulster University, Coleraine, Northern Ireland UK

**Keywords:** GPS, Malaria, Zambia, Population movement

## Abstract

**Background:**

Human movement is a driver of malaria transmission and has implications for sustainable malaria control. However, little research has been done on the impact of fine-scale movement on malaria transmission and control in high-transmission settings. As interest in targeted malaria control increases, evaluations are needed to determine the appropriateness of these strategies in the context of human mobility across a variety of transmission settings.

**Methods:**

A human mobility study was conducted in Nchelenge District, a high-transmission setting in northern Zambia. Over 1 year, 84 participants were recruited from active malaria surveillance cohorts to wear a global positioning system data logger for 1 month during all daily activity. Participants completed a survey questionnaire and underwent malaria testing and treatment at the time of logger distribution and at collection 1 month later. Incident malaria infections were identified using polymerase chain reaction. Participant movement was characterized throughout the study area and across areas targeted for an indoor residual spraying (IRS) intervention. Participant movement patterns were compared using movement intensity maps, activity space plots, and statistical analyses. Malaria risk was characterized across participants using spatial risk maps and time spent away from home during peak vector biting hours.

**Results:**

Movement data were collected from 82 participants, and 63 completed a second study visit. Participants exhibited diverse mobility patterns across the study area, including movement into and out of areas targeted for IRS, potentially mitigating the impact of IRS on parasite prevalence. Movement patterns did not differ significantly by season or age, but male participants traveled longer distances and spent more time away from home. Monthly malaria incidence was 22%, and malaria risk was characterized as high across participants. Participants with incident parasitemia traveled a shorter distance and spent more time away from home during peak biting hours; however, these relationships were not statistically significant, and malaria risk score did not differ by incident parasitemia.

**Conclusions:**

Individual movement patterns in Nchelenge District, Zambia have implications for malaria control, particularly the effectiveness of targeted IRS strategies. Large and fine-scale population mobility patterns should be considered when planning intervention strategies across transmission settings.

**Electronic supplementary material:**

The online version of this article (10.1186/s12942-019-0183-y) contains supplementary material, which is available to authorized users.

## Background

Human movement is an important driver of malaria transmission. Movement of infected individuals between high and low-transmission settings is a source of parasite introductions and can facilitate the spread of drug resistance [1–6]. Large-scale movement across borders and between urban and rural areas can result in importation and reemergence of malaria in areas with previously successful malaria control [3, 7–9]. If competent vectors are present, reintroduction events can lead to a resurgence of malaria transmission in the absence of sustained malaria control programs [9]. These types of patterns have been cited as contributing causes for the failure of the Global Malaria Eradication Program (GMEP) [7, 10]. Due to these factors, malaria control in regions receptive to transmission may be inherently unstable, and accounting for human movement is necessary for sustainable control. Studies in recent years have further strengthened this conclusion using novel methods of characterizing human mobility patterns, including anonymized cellular phone records, census-based migration data, and records of ticketed travel [11–17].

Although the importance of large-scale movement has been documented, little is known about how small-scale movement patterns affect malaria transmission [7]. Studies have demonstrated that malaria transmission varies across fine spatial scales, and that daily movement across areas of differing transmission can increase heterogeneity in individual disease risk [18–24]. This heterogeneity can in turn increase population-level transmission [18–21]. Consequently, individual movement patterns are likely to contribute to both personal and population-level malaria risk. In one modelling study, individual movement patterns during times of active vector biting were a more significant predictor of disease risk than vector density in the individual’s home [19].

Small-scale movement may also impact the effectiveness of malaria control activities. While large-scale movement has been shown to undermine interventions at the national and sub-national levels, mobility between areas of heterogeneous malaria transmission at finer levels of spatial resolution could also be expected to adversely impact the effectiveness of interventions. This is particularly relevant as national malaria control programs advance and interventions become increasingly targeted to transmission hotspots [25–27]. If areas of residual high transmission remain, local mobility between areas of differing malaria transmission could attenuate the impact of interventions through inward movement from neighboring non-intervention areas and outward movement into areas with greater risk of exposure.

Portable global positioning system (GPS) devices are an emerging method to investigate the impact of small-scale movement patterns on disease transmission. These devices can provide spatial data with high confidence and at fine levels of spatial and temporal resolution. Importantly, GPS devices can be linked to individuals, have high acceptability to study participants, and are reliable in rural settings [28–30]. GPS devices have successfully been used to investigate the impact of individual movement on transmission of malaria, dengue, schistosomiasis, filariasis, and hookworm [31–35]. Most notably, recent studies in Peru and southern Zambia used GPS data loggers to collect detailed movement data in resource-poor environments, with findings that helped explain epidemiologic patterns of vector-borne diseases [31, 34].

We conducted a population movement study in Nchelenge District, Luapula Province, Zambia using commercially available GPS data loggers. The aims of this study were to describe human movement patterns in a remote, rural area of sub-Saharan Africa to assess the relationships between movement and malaria risk in a high-transmission setting, and to investigate the potential impact of fine-scale mobility on targeted indoor residual spraying (IRS).

## Methods

### Study site

Nchelenge District is located in Luapula Province in northern Zambia along the banks of Lake Mweru. The population is largely agrarian, with fishing as the main economic activity during the dry season and farming occurring in inland regions during the rainy season when an annual fishing ban is imposed [36]. There is a land border with the Democratic Republic of the Congo (DRC) to the north, across which a large degree of formal and informal movement is presumed to occur.

**Fig. 1:**
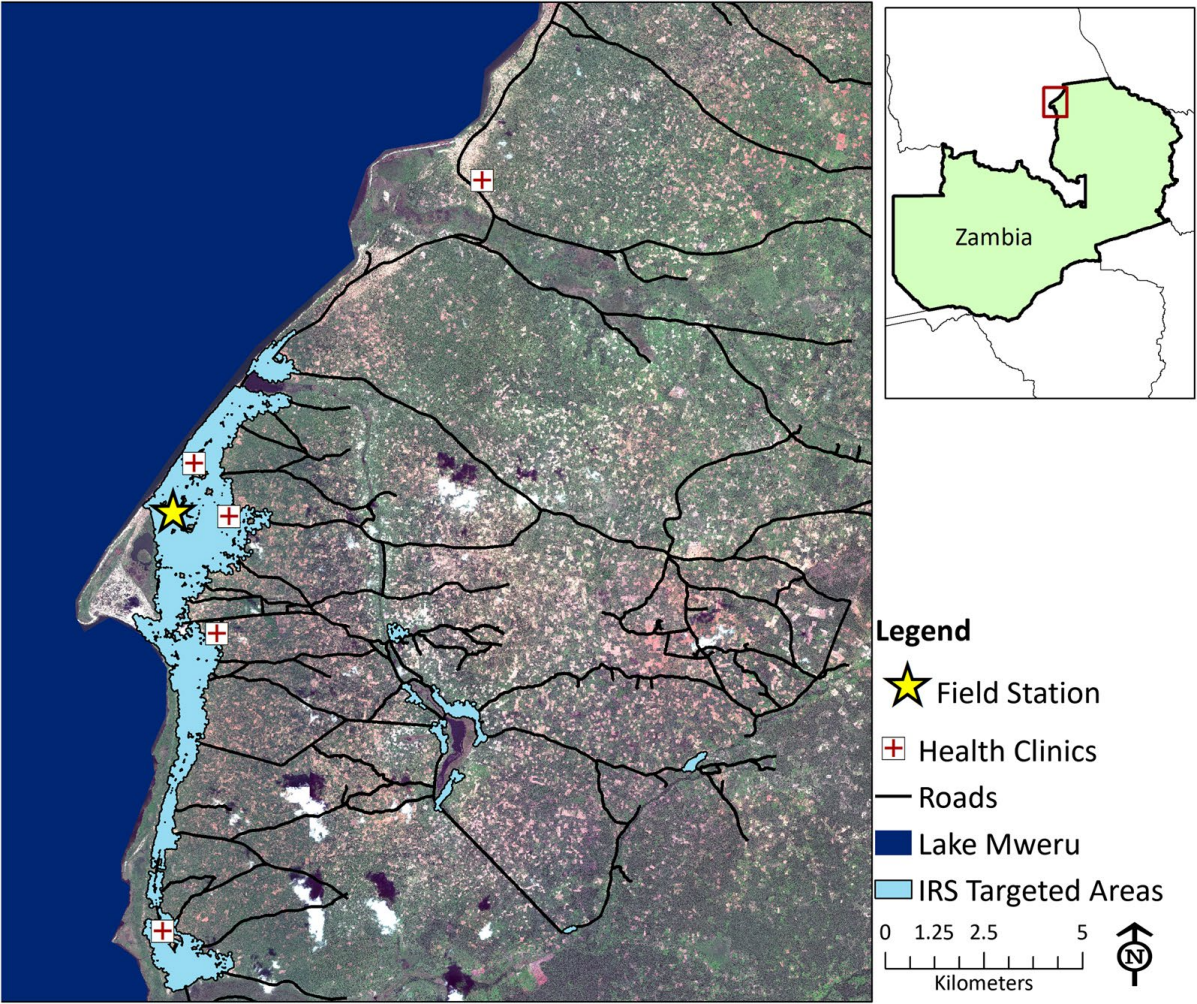
Study area in Nchelenge District, Zambia with areas targeted for IRS highlighted

Malaria transmission in Nchelenge District remains high despite Zambia’s extensive national malaria control strategy, which includes universal access to long-lasting insecticide-treated bed nets (LLINs), rapid diagnostic tests (RDTs), and artemisinin-combination therapy (ACTs). Parasite prevalence by RDT averages nearly 70% in school-age children, and residents receive approximately 140 infective bites per year [37–40]. The marshland environment supports year-round transmission, and high resistance to pyrethroid and carbamate insecticides have been found in the primary malaria vectors, *Anopheles funestus* and *An. gambiae* sensu stricto (s.s.) [36, 37, 41, 42]. In October 2014, the first of a series of annual IRS campaigns was conducted in Nchelenge District using the organophosphate insecticide pirimiphos-methyl [43]. The campaigns used a targeted strategy, and spray activities were focused in the highly-populated peri-urban areas along Lake Mweru calculated to have the highest risk [43] (Fig. 1).

### GPS data loggers

The GPS data loggers were IgotU^®^ GT-600 devices (Mobile Action Technology, New Taipei City, Taiwan). These were selected based on their acceptability for field study, which included ease in programming, light weight (37 grams), large memory (> 250,000 points), long battery life (30 h of continuous use), water resistance, and relatively low cost [29]. Under open sky, they are reported to be accurate within 20 m at least 90% of the time, and they have an average error of 4.4 m while stationary and of 10.3 m while in motion [28, 29]. Data loggers were programmed to be motion-activated to preserve battery life and to record geographic position, date, and time, every 2.5 min while active. Devices were password protected, and data could only be accessed with a specific software program and unique connection cable, which were kept at the locked field station. To reduce errors, the power button was disabled so participants could not accidentally turn off the device.

### Study population

The population for the GPS data logger study was recruited from active surveillance cohorts of the Southern Africa International Centers of Excellence for Malaria Research (ICEMR) [44]. Detailed methods of the Southern Africa ICEMR are described elsewhere [36, 41, 45]. In brief, a 1 × 1 km grid was overlaid over the study area, and grid quadrants were selected using stratified random sampling to obtain a representative sample based on geography and population density. Households were enumerated using Quickbird™ satellite images (DigitalGlobal Services, Denver, CO). Each month, 25–30 households were randomly selected from grid quadrants and were recruited into longitudinal or cross-sectional cohorts. Cross-sectional households were each visited once, and longitudinal households were visited every other month for 1 year. At each study visit, consenting household members completed a survey on demographic characteristics, history of recent malaria and treatment, and history of household malaria interventions. Participants provided blood samples by finger prick for hemoglobin testing, *Plasmodium falciparum* HRP-2 RDT (Standard Diagnostics, Kyonggi, Republic of Korea), and dried blood spots (DBS). Participant temperature was measured using a digital ear thermometer, and household coordinates were recorded on a GPS-enabled handheld tablet. Participants with a positive RDT were offered treatment with Coartem^®^ (Novartis, Basel, Switzerland), the first-line standard of care in Zambia.

### GPS data collection

Sensitization activities for the GPS study occurred in June 2014, coinciding with enrollment of a new longitudinal cohort. New longitudinal households were visited by study teams and were informed of the additional option to participate in the GPS study within the following year. Study procedures were explained, including the purpose of the study and protection of confidentiality, and pamphlets were translated and distributed in Bemba, the local language. Recruitment began in August 2014 and continued every other month until June 2015.

In each recruitment month, a convenience sample of 12–15 participants aged 13 years and older were invited to wear a data logger for approximately 30 days, with no more than two household members participating concurrently. If more than two eligible household members were available in the same month, the study team would select two to participate and then would enroll the remaining eligible household members in a subsequent data collection month. Participants were included from only the longitudinal cohort from August to December 2014. However, due to higher than expected absences among longitudinal participants on the day of the study visit, participants from the cross-sectional cohorts were also invited to participate starting in February 2015 using the same convenience sampling methods.

Participants were instructed to wear the data logger at all times during their normal daily activity, except when bathing or swimming. Devices were worn around the wrist like a watch, on a lanyard around the neck, or in a pocket or bag. To ensure battery life, the data logger was collected after 15 days and replaced with a fully charged device for the remainder of the month. Data logger serial numbers were matched to unique participant ID numbers, and the date and time were recorded at each logger distribution and collection for every participant.

The standard study visit activities, including collection of blood samples and completion of the survey, were conducted at data logger distribution (visit 1) and repeated at the final collection (visit 2), with the aim of having two complete study visits approximately 30 days apart. Because parasitemia confirmed by RDT was identified and treated at visit 1, positive test results at visit 2 were classified as incident malaria infections.

### Laboratory procedures

Filter paper with DBS (Whatman 903™ Protein Saver cards) were dried, sealed in plastic Ziploc bags containing a desiccant, and stored at the field station in Nchelenge District at − 20 °C. They were transported to the laboratory at the Tropical Diseases Research Centre in Ndola, Zambia and stored at −  20 °C. Parasite DNA was recovered using Chelex© extraction within 1 year of sample collection [46]. A nested PCR assay was conducted within 1 month of DNA extraction to detect *Plasmodium* DNA targeting a segment of the mitochondrial cytochrome b gene [47]. Reactions were run in a GeneAmp PCR System 9700 thermal cycler (Applied Biosystems, Foster City, CA). Amplified product was detected by electrophoresis on 1% agarose gel and viewed under UV light as an 815-base pair DNA band [48].

### Data management and processing

Data from returned GPS data loggers were uploaded onto a password-protected computer in the field office using the @trip software developed for use with IgotU products. De-identified files were sent to the data manager at Macha Research Trust, Choma District, Zambia who removed data points not within the appropriate time interval for each participant. Cleaned data logger files and survey data from visits 1 and 2 were uploaded into REDCap secure file-sharing software [49]. Logger data and geolocated household locations were plotted in ArcGIS Version 10.2 (ESRI, Redlands, CA). Logger data were cleaned using a software extension developed for trajectory analysis in ArcGIS, which removes potentially erroneous data points based on improbable speeds or abrupt changes in direction [50]. For each participant, the distances from each logged point to their household, Lake Mweru, the borders of the study area, and areas targeted for IRS were calculated. If the participants had two households due to seasonal agricultural practices, distance was calculated to the closest household.

Images of participant data points across the study area were created in ArcGIS and stratified by rainy and dry season. The rainy season was defined as the months in which average daily rainfall exceeded 1 mm/day, which included October 2014–March 2015. Movement intensity plots were developed by month of data collection and season using the ArcGIS extension software [50]. These images indicate the cumulative time spent in each location using color and height. Data points were projected onto previously created malaria risk maps of the study area, which were developed from the first 2 years of active surveillance data in Nchelenge District from 2012 to 2014 [45]. Separate risk maps were created for the rainy and dry seasons, and movement data points collected in each season were projected onto the appropriate seasonal map. For each geolocated data logger point, the value of the appropriate underlying risk map raster pixel was extracted to obtain a measure of calculated malaria risk for that location, $R_{k}$.

### Data analysis

Processed GPS data were loaded into STATA 13.1 (Stata-Corporation, College Station, TX) and merged by participant ID and date to household survey data for visits 1 and 2. Malaria status at each visit was determined by RDT and PCR results, with positive PCR results at visit 2 considered incident infections. Participants were defined to have a fever if their tympanic temperature exceeded 38 °C, and anemia was determined by WHO criteria for hemoglobin levels by age and sex [51]. Individual and household-level correlates of PCR results at visits 1 and 2 were identified using Chi squared tests. Differences in characteristics between retained participants and participants lost to follow-up were also calculated using Chi squared tests.

For each individual $i$, total participation time $T_{i}$ was calculated, excluding any missing time between the two logger distributions caused by early battery failure or other complications. The amount of time spent at each logged point location $\tau_{ij}$ was estimated to be half the time between the previous and subsequent points. For instance, if points 1, 2, 3, 4, and 5 were recorded at times 7:01, 7:07, 7:23, 7:25, and 7:28 respectively, the amount of time at point 2 would be calculated as 11 min, point 3 would be 9 min, and point 4 would be 2.5 min. The proportion of participant time spent at each logged point location was calculated as the ratio of time spent at that point divided by their total participation time $\left( {{{\tau_{ij} } \mathord{\left/ {\vphantom {{\tau_{ij} } {{\rm T}_{i} }}} \right. \kern-0pt} {{\rm T}_{i} }}} \right)$. Similarly, participation time during peak vector biting hours $\overline{T}_{i}$ was calculated as all recorded time between 6 p.m. and 6 a.m. [52–54], and the proportion of peak biting time at each location was calculated correspondingly as $\left( {{{\tau_{ij} } \mathord{\left/ {\vphantom {{\tau_{ij} } {{\rm T}_{i} }}} \right. \kern-0pt} {{\overline T}_{i} }}} \right)$.

For each participant, the distance between consecutive GPS points was calculated using geodetic distances, or the length of the curve between two points modeled on the surface of the earth [55]. For each participant, the total distance, maximum distance, and average daily distance traveled were calculated for their overall time $T_{i}$ and for peak biting hours $\overline{T}_{i}$. Participants were classified to be at home if they were within 50 m of their household, which was determined to be the distance that most accurately captured movement patterns in sensitivity analyses. Due to limitations in the precision of GPS devices, it could not be confirmed whether participants were inside or directly outside their home.

Based on observed differences in population density and malaria transmission patterns [36, 39, 45], participants were classified to be in the peri-urban lakeside area if they were within 3 km of Lake Mweru and to be in the rural inland area if they were further than 3 km from the lake (Additional file 1: Figure S1). The proportion of time spent at home, at the lakeside, and within areas targeted for IRS were calculated for each participant for both total time $T_{i}$ and peak biting hours $\overline{T}_{i}$. These values were stratified by sex, age category, and parasite positivity at visits 1 and 2, and were compared using Wilcoxon rank-sum tests to account for non-normality of the data. For these comparisons, participants aged 13–17 years were classified as adolescents and participants aged 18 years and older were classified as adults.

### Activity space

To visually portray participant activity space, plots were created of the cumulative proportion of time spent at increasing distances from the household and lakeside, stratified by sex, age category, season, and incident parasitemia at visit 2. These activity space plots were examined at the full spatial extent of distance traveled to compare patterns of large-scale movement and within 1 km of the household to compare patterns of fine-scale movement during peak biting hours.

### Malaria risk

Using previously generated raster maps of malaria risk by season (rainy vs. dry) [56], a risk value $R_{k}$ was extracted for each logged point for the appropriate season. Each individual was then assigned a risk score $R_{i}$, calculated by multiplying the time spent at each logged point by the extracted risk value for that point, summing these risks over the time of individual’s participation, and dividing by the number of days contributed:

$$R_{i} = \frac{{\mathop \sum \nolimits_{j = 1}^{n} \left( {\tau_{ij} *R_{k} } \right)}}{\rm T}$$

This value was calculated as an average daily risk to control for different participation times per person. Average nightly risk $\overline{R}_{i}$ was calculated similarly, using total participation time during peak biting hours $\overline{T}_{i}$. To help account for the impact of malaria control interventions, including use of LLINs and IRS, outdoor nightly risk $\overline{\overline{R}}_{i}$ was also calculated for the total time spent farther than 50 m from the participant’s household during peak biting hours $\overline{\overline{T}}_{i}$. Time outside the study area was censored due to lack of information on malaria risk. The study area was also divided into deciles of malaria risk by rainy and dry season based on the range of values in the original risk maps, and the proportion of time spent at each decile of risk was calculated for each participant. Participant average daily risk, average nightly risk, and average outdoor nightly risk were compared by demographic factors and malaria parasitemia at visits 1 and 2 using Wilcoxon rank-sum tests.

## Results

### Participant characteristics at baseline

**Fig. 2:**
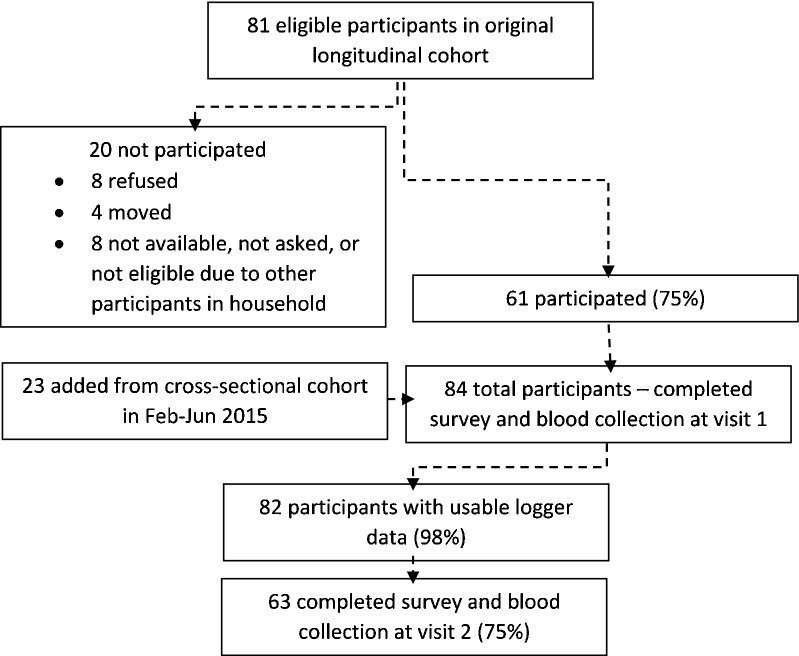
Flowchart of participation

Over six recruitment periods from August 2014 to June 2015, 84 participants were enrolled from 44 unique households. Sixty-one participants were recruited from longitudinal households and 23 were recruited from cross-sectional households (Fig. 2). All available participants in longitudinal households were invited to participate, and at least one participant was enrolled from each longitudinal household. Acceptability of the study was high, with only 10% refusals. The median age at visit 1 was 33 years (range = 13–72 years). Thirty-seven percent of participants were male, 57% lived in lakeside areas, and 41% lived within a 30-min walk from a health clinic, defined as 2.5 km (Table 1). Nearly 40% reported residing in another household for part of the year, with the most common reason being farming (80%). More than half of participants were anemic, 4% had a fever at the time of the study visit, and 28% reported having a fever in the past 2 weeks. Over half reported a visit to a health center in the past month. Coverage of malaria interventions was mixed, with 86% of participants reporting sleeping under a bed net, and 19% reporting a history of household IRS with pirimiphos-methyl in the past year.

**Table 1:** Participant characteristics at visit 1 (N = 84)

	n	%
*Demographics*		
Age < 18	12	14.3
Male	31	36.9
*Household visit type*		
First study visit	40	47.6
Follow-up visit	44	52.4
*Household characteristics*		
Within 3 km of lakeside	49	58.3
Within 30-min walk of health clinic^a^	34	40.5
Reside in different home part of year	31	36.9
Head of household permanently employed	6	7.1
Household uses open water source	37	44.1
Household has dirt floor	81	96.4
*Health-related behaviors*		
Sleeps under bed net	72	85.7
House sprayed with pirimiphos-methyl	16	19.3
Visited health center in past 6 months	47	56.0
Slept away from home in past month	1	1.2
*Clinical results*		
RDT positive	29	34.5
PCR positive	43	51.2
Fever at visit	3	3.6
Report fever in past 2 weeks	23	27.7
Anemic at visit	46	55.4

^a^2.5 km

At visit 1, 35% of participants tested positive for malaria by RDT and 51% tested positive by PCR. Individuals with a positive PCR were more likely to live in inland areas, to live more than 2.5 km (30 min) from a health center, and to use an unprotected water source, but were less likely to have a history of household IRS with pirimiphos-methyl (Table 2). Although not statistically significant, individuals with a positive PCR were frequently younger than 18 years of age.

**Table 2:** Bivariate comparisons of PCR status by participant characteristics at visits 1 and 2

	Visit 1			Visit 2		
	PCR+ n (%) N = 43	PCR− n (%) N = 40	OR (95% CI)	PCR+ n (%) N = 14	PCR− n (%) N = 49	OR (95% CI)
*Age*						
Age < 18	9 (75.0%)	3 (25.0%)	3.2 (0.8, 13.1)	2 (25.0%)	6 (75.0%)	1.2 (0.2, 6.8)
Age ≥ 18	34 (47.9%)	37 (52.1%)	ref	12 (21.4%)	44 (78.6%)	ref
*Sex*						
Male	16 (53.3%)	14 (46.7%)	1.1 (0.5, 2.7)	6 (25.0%)	18 (75.0%)	1.3 (0.4, 4.3)
Female	27 (50.9%)	26 (49.1%)	ref	8 (20.5%)	31 (79.5%)	ref
*Household visit type*						
First ICEMR visit	25 (62.5%)	15 (37.5%)	2.3 (0.96, 5.6)			
Follow-up visit	18 (41.9%)	25 (58.1%)	ref	14 (22.2%)	49 (77.8%)	–
*Distance to lakeside*						
Lakeside < 3 km	20 (40.8%)	29 (59.2%)	0.3 (0.1, 0.8)*	9 (22.5%)	31 (77.5%)	1.0 (0.3, 3.6)
Lakeside ≥ 3 km	23 (67.7%)	11 (32.4%)	ref	5 (21.7%)	18 (78.3%)	ref
*Distance to health center*						
Health center < 30-min walk	10 (29.4%)	24 (70.6%)	0.2 (0.08, 0.5)***	7 (24.1%)	22 (75.9%)	1.2 (0.4, 4.0)
Health center ≥ 30-min walk	33 (67.4%)	16 (32.6%)	ref	7 (20.6%)	27 (79.4%)	ref
*Reside in different home part of year*						
Yes	15 (50.0%)	15 (50.0%)	0.9 (0.3, 2.2)	2 (9.1%)	20 (90.9%)	0.2 (0.05, 1.2)
No	28 (52.8%)	25 (47.2%)	ref	12 (29.3%)	29 (70.7%)	ref
*Household uses open water source*						
Yes	25 (67.6%)	12 (32.4%)	3.2 (1.3, 8.0)**	7 (30.4%)	16 (69.6%)	2.0 (0.6, 6.7)
No	18 (39.1%)	28 (60.9%)	ref	7 (18.0%)	32 (82.0%)	ref
*Sleeps under bed net*						
Yes	36 (50.7%)	35 (49.3%)	0.7 (0.2, 2.5)	11 (22.0%)	39 (78.0%)	0.9 (0.2, 4.0)
No	7 (58.3%)	5 (41.7%)	ref	3 (23.1%)	10 (76.9%)	ref
*House sprayed with actellic*						
Yes	3 (18.8%)	13 (81.2%)	0.2 (0.04, 0.6)**	2 (20.0%)	8 (80.0%)	0.9 (0.2, 5.0)
No	39 (59.1%)	27 (40.9%)	ref	11 (21.2%)	41 (78.8%)	ref

* Chi squared P < 0.05, **P < 0.01 ***P < 0.001

### Malaria incidence

Sixty-three participants completed a second visit, for a loss to follow-up of 25%. Based on demographic and clinical variables measured at visit 1, there were no significant differences between participants who completed the second visit and those who did not. At visit 2, 38% of participants tested positive for parasitemia by RDT and 22% tested positive by PCR (Table 2). Using PCR testing as the gold standard, the 22% who were PCR positive at follow-up were considered to have incident malaria infections. There were no statistically significant differences between participants with and without incident parasitemia at visit 2 (Table 2), however, confidence intervals were wide due to the small sample size.

Among participants who were PCR positive at visit 2, two participants were PCR positive at baseline but were not treated due to a negative RDT. Excluding these participants, incident parasitemia was 20%. All participants who were PCR positive at follow-up were included in further analyses, but sensitivity testing was conducted at each step to ensure that inclusion of these two cases did not influence inferences.

### Participant movement patterns

Data loggers with usable movement tracks were collected from 82 participants (98%). Tracks were collected from participants without a second visit if the device was left at their home or returned by a family member. A total of 181,113 GPS data points were collected, and 99.1% of these were retained following data cleaning. The final clean dataset consisted of 179,443 points, comprising 2407 days of data and covering a combined distance of 11,456 km.

Across all participants, there was a median of 2.7 min (IQR: 2.5–5.4) and a median of 20.9 meters (IQR: 10.1–53.3) between recorded points. Based on the time and distance between points, 1.1% of points were recorded at speeds greater than a typical human running pace (4.5 m/s), indicating that the participant may have been in a vehicle. Individual participants contributed a median of 1882 GPS points (IQR = 888–2989; range = 101–6388) and 31.1 days of data (IQR = 25.0–34.0; range = 9.9–48.4). Total distance traveled per participant ranged from 2.1 to 1169.0 km (median = 91.8; IQR = 38.6–185.2), average daily distance ranged from 0.1 to 42.7 km/day (median = 3.1; IQR = 1.6–6.9), and maximum distance from home ranged from 0.1 to 212.4 km (median = 4.2; IQR = 1.9–10.1) (Additional file 1: Figure S2).

Participants spent a median of 4.7 h away from home per day (IQR = 1.9–8.4; range = 0.1–23.9), and a median of 1.6 h away per night during peak biting hours (IQR = 0.6–4.3; range = 0.01–12.0). Forty-six participants (56%) spent at least 10% of peak biting time away from home. Five participants averaged > 20 h away from home per day and > 11 h away from home per night, suggesting they had a second household that was unknown to the study team.

**Fig. 3:**
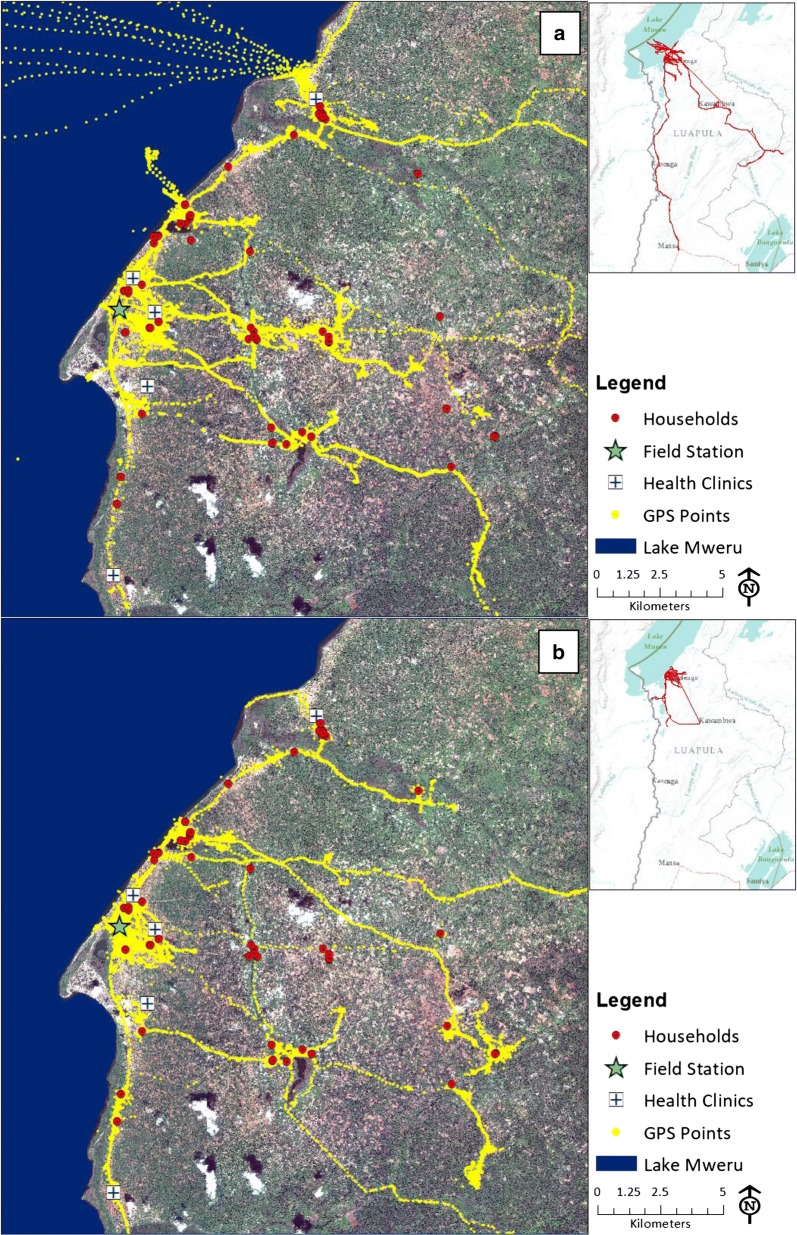
GPS data logger points stratified by **a** dry season and **b** rainy season. Boat travel on Lake Mweru is visible for two participants during the dry season

Participant movement occurred broadly throughout the study area (Fig. 3). Forty participants (49%) spent time in both lakeside and inland areas and made 1–19 round trips between them (median = 3). Thirty (37%) spent time in both lakeside and inland areas during peak biting hours. Similarly, 59 participants (72%) traveled between sprayed and unsprayed areas and made 2–144 round trips between them (median = 17). Thirty-eight (46%) traveled between sprayed and unsprayed areas during peak biting hours. Participants who lived in sprayed areas were more likely to travel between sprayed and unsprayed areas both overall (P = 0.01) and during peak biting times (P = 0.008).

Eight participants (10%) made trips outside Nchelenge District (Fig. 3 inset), and at least one trip was made in each sampled month except February. No participants left Luapula Province and none traveled to a neighboring country. Time outside the district borders ranged from 3 h to 17 days, and five participants spent over 24 cumulative hours outside the district. The number of trips outside the district ranged from 1 to 14 (median = 4). The two persons with the highest number of trips were fishermen, who left the borders of the district 14 and 7 times, respectively, to go onto the lake in boats (Fig. 3a). By distance, 21 participants traveled at least 10 km away from their homes, 9 traveled at least 20 km, 5 traveled at least 30 km, and 2 traveled over 100 km.

In participant surveys, 8 participants reported sleeping away from home between visits 1 and 2, all of whom reported sleeping in the homes of friends or family while away. Three participants traveled for work, two visited friends or family, two traveled for funerals, and one traveled to buy or sell goods. Trips lasted 1–14 nights (median = 7). Six participants reported staying in another village in the district and two reported staying in another district within Luapula Province. These reports do not match the GPS data exactly because four of eight participants who left Nchelenge District were lost to follow up.

### Seasonal movement patterns

**Fig. 4:**
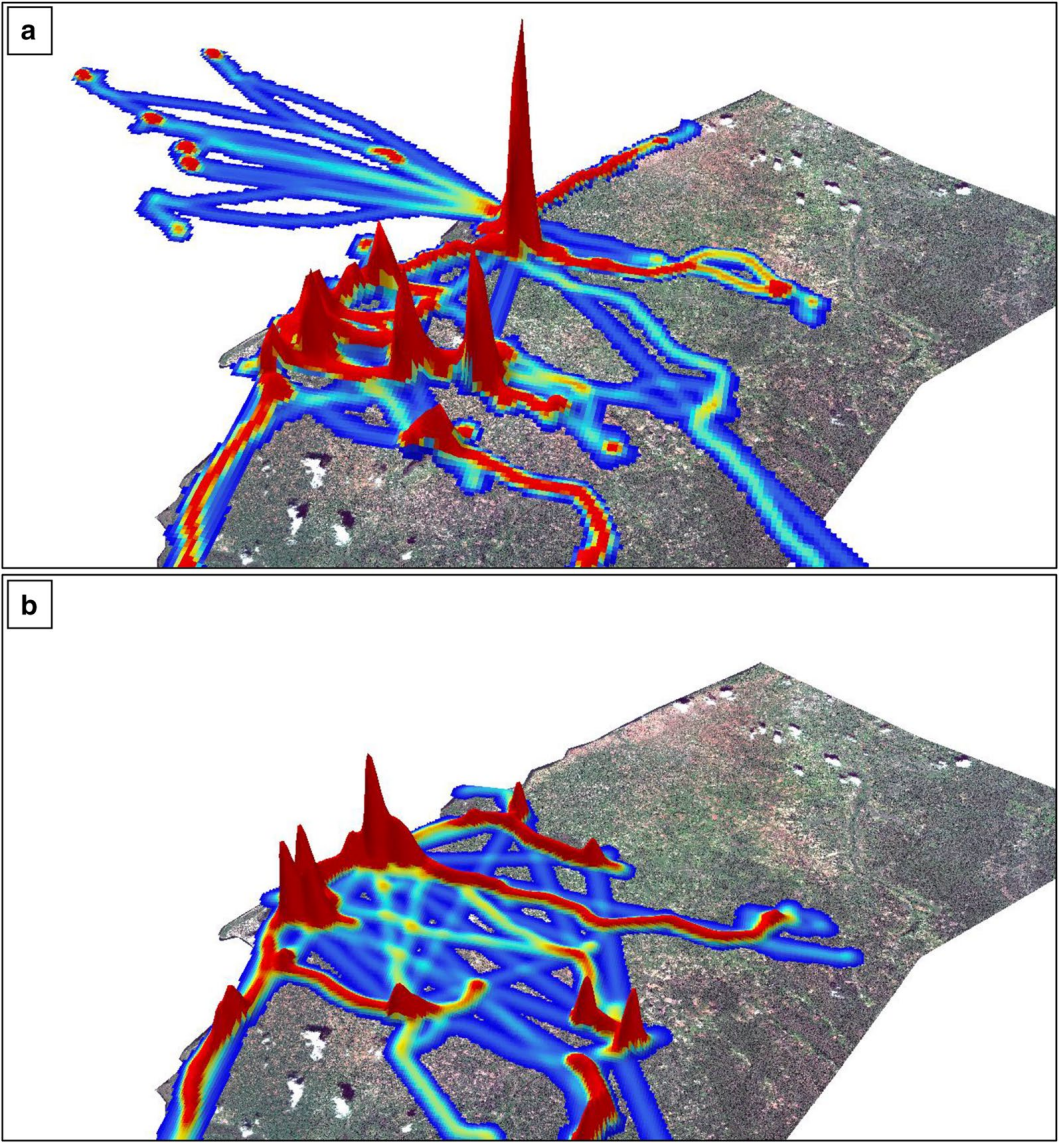
Three-dimensional intensity maps of population movement in Nchelenge District in **a** dry season, and **b** rainy season

**Fig. 5:**
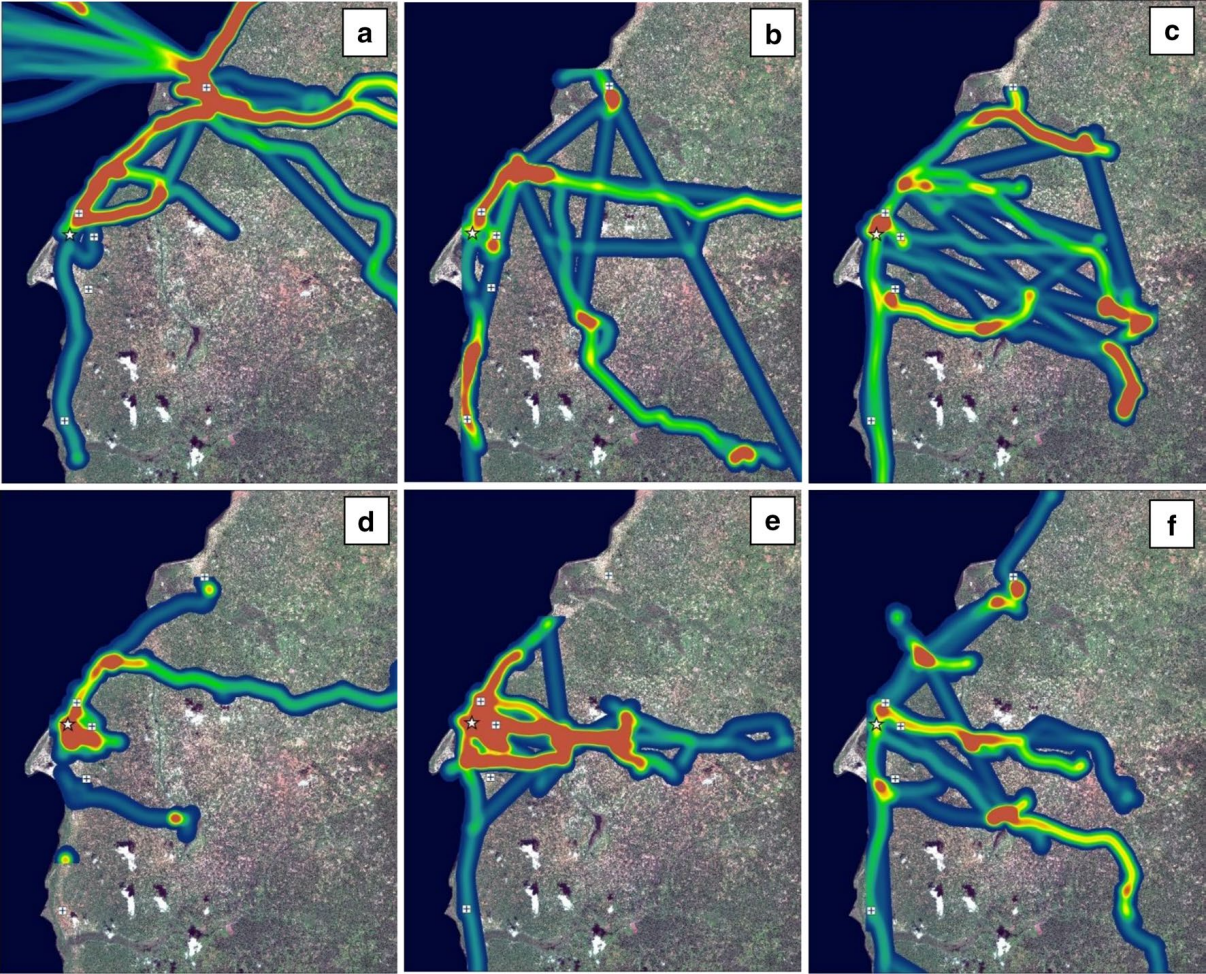
Intensity maps of population movement in Nchelenge District from August 2014 to June 2015 by month in **a** August 2014, **b** October 2014, **c** December 2014, **d** February 2015, **e** April 2015, and **f** June 2015. Boat travel on Lake Mweru is visible in panels **a** and **f**

Contrary to expectations, movement patterns in this region were not clearly seasonal (Fig. 3). Due to agricultural practices, it was anticipated that movement would concentrate along Lake Mweru during the fishing period in the dry season but would concentrate in the inland area during the rainy season while the fishing ban was in effect. Fishing activity was observed during the dry season (Fig. 3a), but participants frequently moved throughout the study area during both seasons, and participants with two known households went back and forth between them throughout the year. Participants traveled outside Nchelenge District in both dry and rainy seasons, although participants traveled longer distances in the dry season (Fig. 3a, b insets). In movement intensity plots (Figs. 4, 5), most participant time throughout the year was spent in the high-population density area near the lake, but local maxima were observed across the study area in all months and seasons. In these plots, straight line segments indicate high speeds and thus longer distances between points, for example travel in a car or bus. This type of travel was also observed throughout the year.

**Fig. 6:**
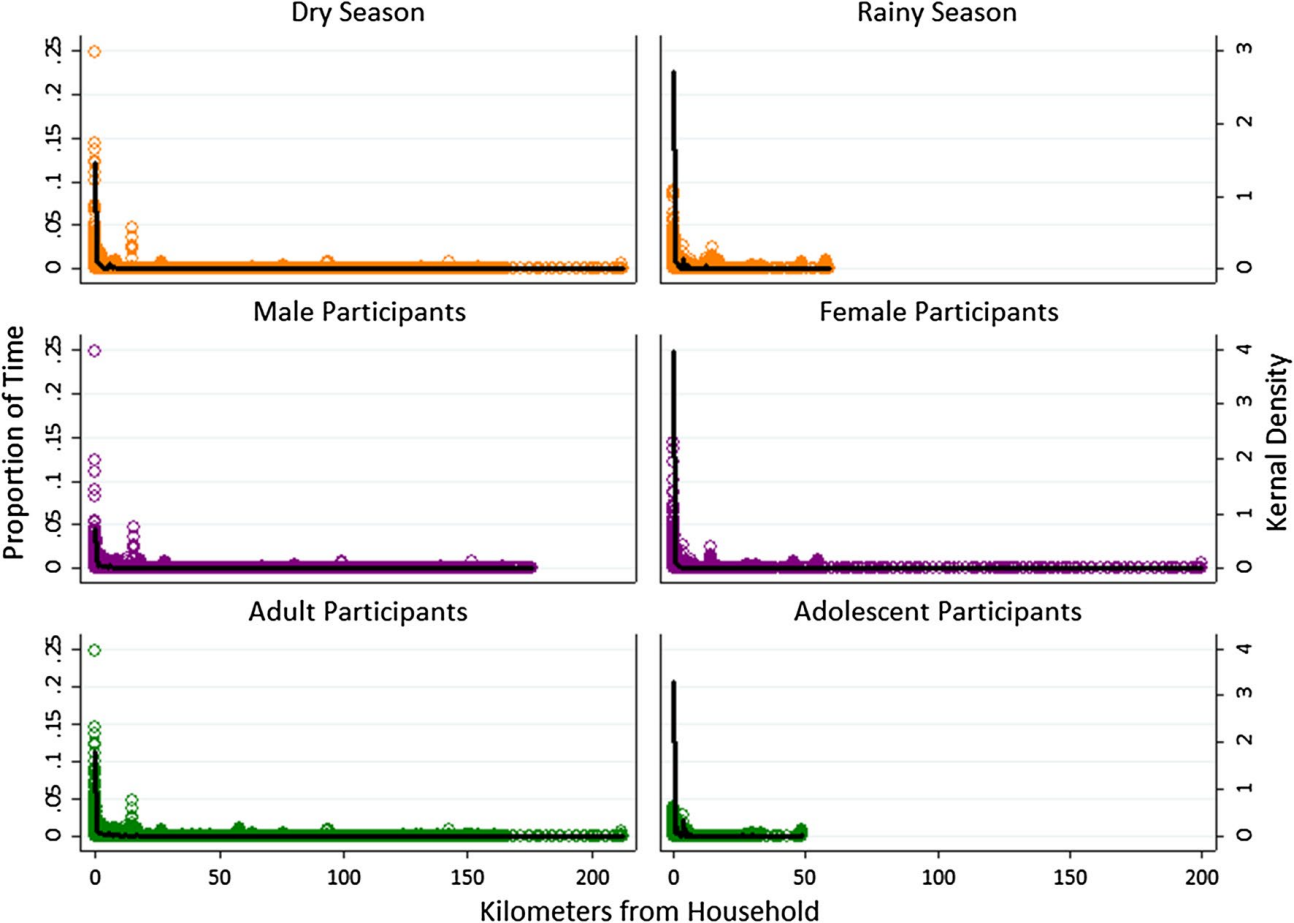
Activity space plots for participants showing the proportion of time spent by distance from participant household, stratified by season of participation, sex, and age

**Fig. 7:**
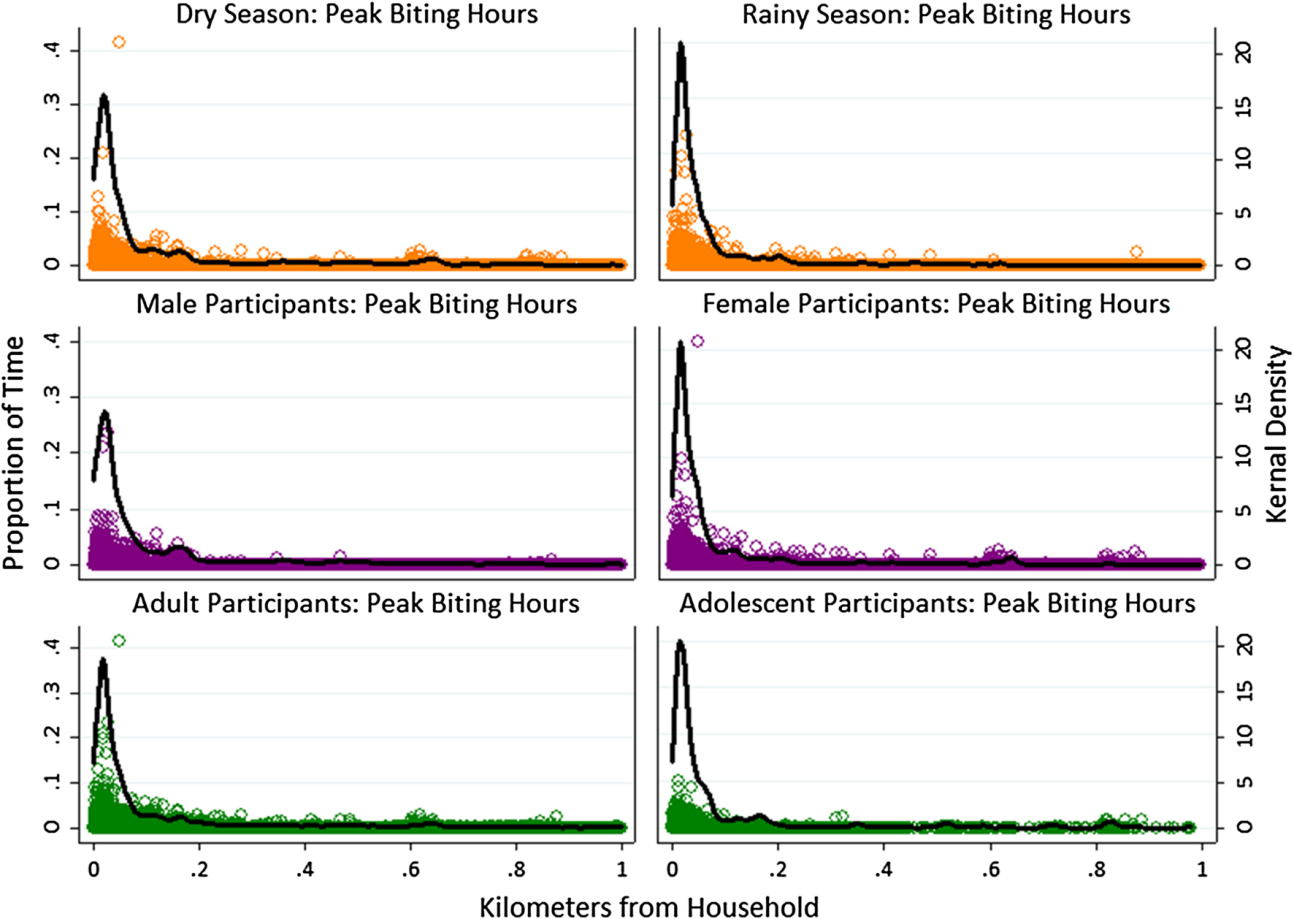
Activity space plots for participants showing the proportion of peak vector biting time spent by distance from participant household, stratified by season of participation, sex, and age

No significant differences were found between seasons for the number of points recorded, total time contributed, total or maximum distance traveled, or average hours spent away from home, for either total time or peak biting hours. However, activity space plots visually show that participants traveled a longer distance in the dry season, as shown by a longer tail, and spent a larger proportion of time near home in the rainy season, as shown by a higher peak at short distances and a thinner tail (Figs. 6, 7).

### Movement patterns by sex and age

Male participants contributed a higher mean number of points than female participants (P = 0.008), but not a higher mean number of days. Male participants also traveled longer total distances, longer distances per day, traveled farther from home, and spent more hours away from home during both total time and peak biting hours (Table 3). Male participants were more likely to travel between lakeside and inland areas (P = 0.05) but were not more likely to travel between sprayed and unsprayed areas. Male participants were also more likely to travel more than 10 km away from home (P = 0.005), but there was no difference at longer distances. There were no significant differences in these metrics by age.

**Table 3:** Metrics of movement patterns among participants, stratified by sex, PCR positivity at the second visit, and peak vector biting hours

	Males		Females		P value*
	Median (IQR)	Range	Median (IQR)	Range	
*Overall*					
Total distance traveled (km)	145.0 (58.3–255.5)	14.0–1169.0	79.7 (28.9–158.2)	2.1–511.8	*0.01*
Average distance per day (km)	5.9 (2.4–8.9)	0.7–42.7	2.4 (1.0–4.5)	0.1–15.5	*0.003*
Maximum distance from home (km)	8.7 (2.0–14.1)	0.2–165.9	3.2 (1.5–7.1)	0.1–212.4	*0.03*
Average hours away from home per day (> 50 m)	6.4 (2.6–14.3)	0.5–23.9	3.8 (1.4–6.5)	0.1–23.8	*0.02*
*Peak biting hours* ^a^					
Total distance traveled	26.8 (14.2–42.7)	1.9–174.8	17.3 (7.9–34.1)	0.9–74.8	*0.04*
Average distance per day	2.4 (1.3–3.1)	0.2–11.7	1.4 (0.6–2.2)	0.1–9.2	*0.01*
Maximum distance from home	2.4 (0.6–9.2)	0.06–154.9	1.7 (0.4–3.4)	0.07–212.4	0.2
Average hours away from home per night (> 50 m)	2.9 (0.9–6.3)	0.01–12.0	1.3 (0.5–2.5)	0.1–12.0	0.06

	PCR+		PCR−		P value*
	Median (IQR)	Range	Median (IQR)	Range	
*Overall*					
Total distance traveled (km)	78.3 (28.2–130.0)	7.4–346.1	88.2 (52.8–181.7)	6.7–1169.0	0.4
Average distance per day (km)	3.0 (1.2–3.9)	0.3–10.1	2.8 (1.8 - 6.5)	0.3–42.7	0.4
Maximum distance from home (km)	4.2 (2.3–8.0)	0.3–16.4	3.7 (1.9–8.8)	0.2–212.4	0.9
Average hours away from home per day (> 50 m)	2.6 (1.3–6.4)	0.5–10.0	4.5 (2.3–6.8)	0.8–23.9	0.3
*Peak biting hours* ^a^					
Total distance traveled	15.9 (8.3–30.1)	2.2–58.4	21.6 (10.1–37.0)	1.9–174.8	0.7
Average distance per day	1.8 (0.6–2.2)	0.2–3.9	1.8 (0.8–2.6)	0.2–11.7	0.6
Maximum distance from home	2.2 (0.7–4.0)	0.1–9.2	1.8 (0.6–4.0)	0.1–212.4	0.7
Average hours away from home per night (> 50 m)	1.0 (0.5–2.7)	0.2–5.0	1.6 (0.8–2.8)	0.01–12.0	0.5

* Wilcoxon rank sum test

^a^Peak biting hours from 6 p.m. to 6 a.m.

Similarly, in activity space plots, the maximum distance traveled was similar between males and female participants, but female participants spent a greater proportion of both overall and peak biting time near their home (Fig. 6, 7). Adult participants traveled further and spent less total time near their home than adolescents younger than 18 years (Figs. 6), but both age groups spent a similar proportion of time near their home during peak biting hours (Fig. 7).

### Movement patterns and malaria incidence

Movement patterns were not significantly associated with incident parasitemia in this population. No significant differences were found between PCR positive and negative participants at visit 2 for number of points or time contributed, total or maximum distance traveled, or average hours spent away from home, for either total time or peak biting hours (Table 3). There was no significant difference in travel between lakeside and inland areas, between sprayed and unsprayed areas, or to various distances from the home.

**Fig. 8:**
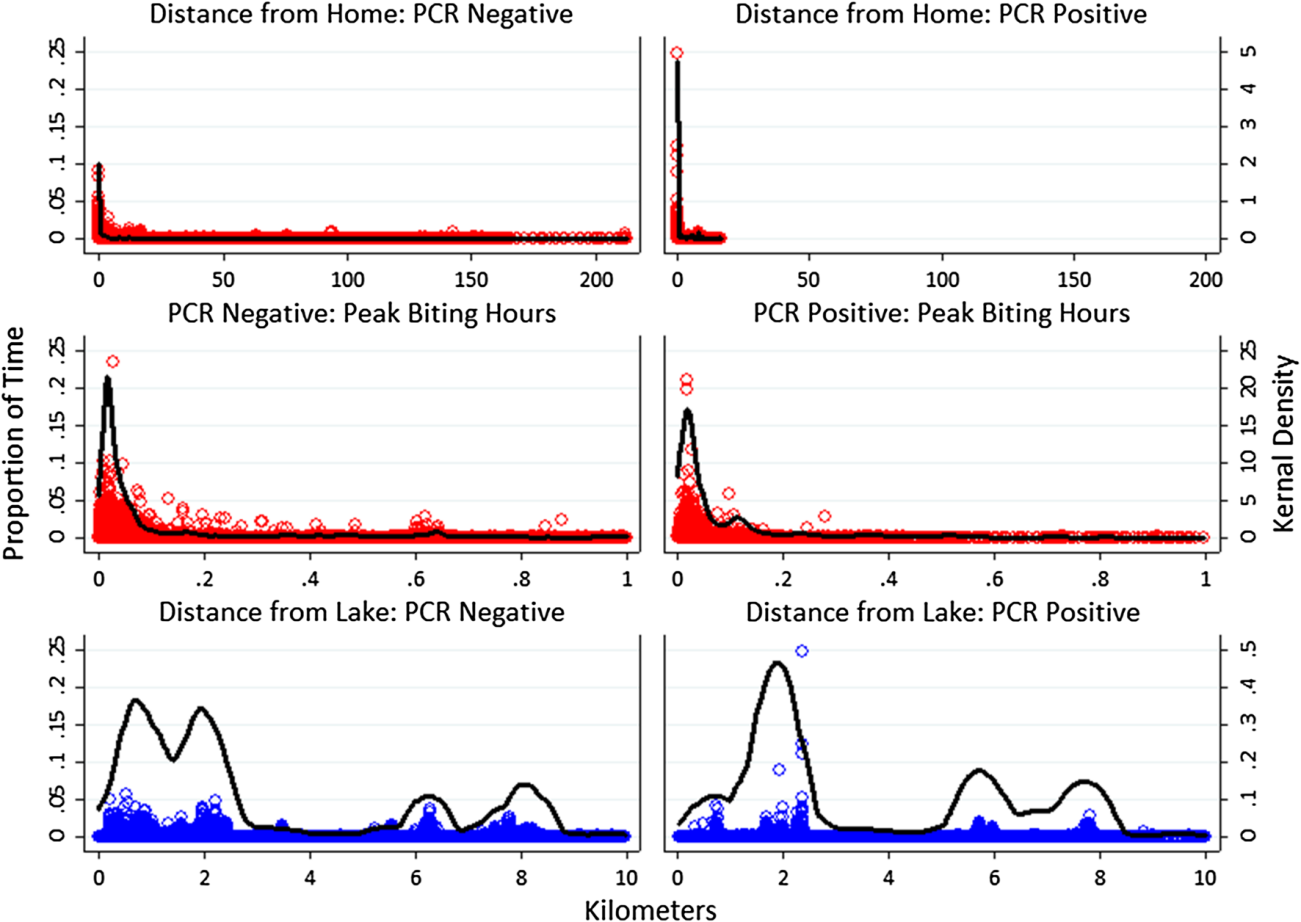
Activity space plots for participants showing the proportion of time spent by distance from participant household or Lake Mweru, stratified by season of PCR positivity at the second visit

Although not statistically significant, several patterns were evident in activity space plots (Fig. 8). Participants with incident parasitemia traveled shorter maximum distances, and all participants who traveled at least 20 km away from home were PCR negative at visit 2. Conversely, participants with incident parasitemia spent fewer peak biting hours at home and therefore spent more time outside the home during these hours. Participants with incident parasitemia also spent less time within 1 km of the lakeside and spent more time greater than 5 km from the lake.

### Movement patterns and malaria risk

Participant movement was largely concentrated in areas at highest risk for malaria (Additional file 1: Figure S3). Approximately 12% of all participant time was spent in areas within the top decile of malaria risk, 34% of time was in the top three deciles, and 72% of time was spent above the median of malaria risk. These values were consistent during peak biting hours and across demographic groups. There were no significant differences in individual daily, nightly, or outdoor nightly risk score by PCR positivity at either visits 1 or 2 (Additional file 1: Figure S4). In general, malaria risk was determined to be high for all participant movement patterns in this high transmission setting.

## Discussion

In this high malaria transmission setting in northern Zambia, participants exhibited a high degree of mobility at both small- and large-scales throughout the year. A large proportion of time was spent near the home and in the densely populated area near the lake, including in areas targeted for IRS with pirimiphos-methyl. One-third of participants resided in multiple homes, and movement between homes was common throughout the year. Mobility was less seasonal than anticipated, with both intra-district movement and long-distance travel occurring with similar frequency throughout the year despite the annual fishing ban and the occurrence of flooding in the rainy season. During their month of data collection, half of participants traveled between the lakeside and inland areas, nearly three quarters traveled between sprayed and unsprayed areas, and one in ten traveled outside the district. During peak biting hours, one-third of participants traveled between lakeside and inland areas, and nearly half traveled between sprayed and unsprayed areas. Male and adult participants spent more time away from home during peak biting hours, and males exhibited higher overall nighttime mobility. Participants spent nearly three-quarters of peak biting time in areas predicted to be at high risk for malaria.

These movement patterns have implications for malaria control. In particular, the frequency of movement in and out of sprayed areas could attenuate the impact of the targeted IRS intervention through reintroductions and exposures. In addition, the presence of multiple households and lack of firm seasonal residence patterns may undermine malaria control efforts if people are not reliably at a particular home to consent to and receive interventions. The substantial proportion of peak biting time spent away from home may further reduce the efficacy of indoor vector control interventions. Given the high vector density in this region [36, 41] and reports of outdoor biting in other locations [54, 57–60], time spent outside at night is likely to contribute to malaria transmission in Nchelenge District. The observed value of 10% is also likely an underestimate due to limitations in the precision of the GPS devices, so the true time spent outside at night is likely higher than estimated here. Given these factors, the movement patterns observed in Nchelenge District may reduce the effectiveness of a focal IRS strategy and highlight the need to better understand human mobility when planning malaria control interventions.

In this population, there was a 22% monthly malaria incidence by PCR among participants with a second visit. For a short period of follow-up, this indicates a very high level of transmission. Different movement patterns were expected to emerge among those with or without incident malaria; however, few clear relationships were evident. In activity space plots, participants with incident parasitemia spent more time away from home during peak biting hours and did not travel more than 20 km, but these relationships were not statistically significant. There are several potential explanations for this result. Due to the small sample size and short follow-up time, only 14 participants were PCR positive at visit 2, which resulted in wide confidence intervals and limited power to draw inferences between groups. Therefore, true relationships may exist that would be observable with a larger study population. Alternatively, these analyses may reflect a true absence of a relationship, perhaps due to lack of variation between participants or saturation of malaria risk. If transmission is sufficiently high, movement patterns may not emerge as a significant predictor of individual risk.

This study had several limitations. The sample size was not sufficiently large to conduct all statistical comparisons with precision, and loss to follow-up for the second visit was 25%. Participants who were lost to follow-up did not differ by demographic or clinical characteristics at baseline, but their movement data indicated that they were more likely to travel longer distances, so they may not be fully comparable. A larger sample size and greater retention would have improved the inferential power.

The use of non-probability sampling and the restriction to adolescents and adults may also limit the representativeness of this study. Adolescents and adult men were under-sampled compared to the overall population, and children under 13 years were excluded due to concerns about their reliability in carrying the devices, so the study population may not fully represent the underlying population at greatest risk. Young children’s movement patterns may be expected to mirror that of their mothers [61], but this assumption may not be valid for school-age children. Future studies would benefit from including more adult men, adolescents, and young children to determine if relevant differences emerge.

Another limitation was the potential for inaccuracies in the GPS logger data caused by imprecision and user error. Due to lower indoor accuracy of the GPS devices, sensitivity analyses indicated that participants should be classified as being at home if they were within 50 m of their household. Therefore, participants could not be reliably categorized as inside or outside. Total distance traveled may also be underestimated due to the assumption of linear movement, and GPS data may have been incorrect if the logger was forgotten at home, intentionally not used, or used by another person. Since these scenarios were difficult to verify, logger data was analyzed as collected unless a specific issue was reported to the field team, and compliance was therefore challenging to assess.

A final concern was the high level of discordance between RDT and PCR results. At visit 1, RDT results underestimated true parasite prevalence by PCR by 31%, and several PCR positive individuals were not treated at their first visit. Although 80% of these infections resolved by visit 2, two participants were RDT negative but PCR positive at visit 1 and remained PCR positive at visit 2, so it is unknown if these were new or persistent infections. If these two people were excluded, the monthly incidence was 20%. Sensitivity analyses were conducted to ensure that this did not impact inferences. These false negatives are likely due to low-level parasitemia since HRP-2 deletions have not been found in Nchelenge District [62]. Conversely, RDT results overestimated malaria incidence by 73% at visit 2. Several studies have demonstrated false positive RDTs after parasite clearance due to persistence of HRP-2 antigen, and therefore a 1 month interval may not be long enough to accurately detect incident infections with RDTs in this high-transmission setting [63–66]. PCR results were therefore used for all analyses involving malaria incidence.

Despite these limitations, this study had considerable strengths. The project extended research conducted in Peru and Southern Zambia, but to our knowledge was the first to attempt to link movement patterns with incident malaria parasitemia through a second study visit. Although loss to follow up occurred, incident malaria infection was identified in 1 of 5 participants, indicating that this study design is feasible in high-transmission settings. Despite the small sample size, a large amount of geolocated data was collected over a year in a challenging research setting. High-burden rural populations in sub-Saharan Africa are often difficult to access for logistical or political reasons, making this dataset unusual among human movement studies. Moreover, a wide variety of movement patterns were captured with direct implications for targeted malaria control. High acceptability was observed among study participants, suggesting that a larger sample size or longer follow-up would be feasible.

## Conclusion

Population movement has significant implications for malaria control at both large and small spatial scales. Over 1 month of participation in a GPS data logger study, residents of Nchelenge District, Zambia spent a large proportion of time in high-risk areas and exhibited a wide range of movement patterns. These behaviors can increase individual malaria exposures, amplify population-level transmission, and impact malaria control efforts. In this high-transmission setting, movement patterns likely attenuated the impact of a targeted IRS strategy due to frequent movement in and out of sprayed areas. This is the first time that fine-scale movement data has been directly linked to malaria incidence, and although there was insufficient power to conclusively draw inferences at the individual level, this study provides evidence of the importance of individual movement patterns for malaria transmission and the feasibility of similar investigations to inform malaria control policy. Overall, human mobility should be considered when selecting intervention strategies for similar high-transmission settings, particularly when designing targeted control strategies.

## Additional file

**Additional file 1.** Additional figures.

## Data Availability

The datasets generated and/or analyzed in this study are not publicly available due to privacy concerns for participants but are available from the corresponding author on reasonable request.

## Abbreviations

GPS: global positioning system
PCR: polymerase chain reaction
IRS: indoor residual spraying
GMEP: global malaria eradication program
DRC: Democratic Republic of the Congo
LLIN: long-lasting insecticide treated net
RDT: rapid diagnostic test
ACT: artemisinin combination therapy
ICEMR: International Centers of Excellence in Malaria Research
