# Supplementary material for: The use of GPS data loggers to describe the impact of spatio-temporal movement patterns on malaria control in a high-transmission area of northern Zambia
Source: Int J Health Geogr. 2019 Aug 19;18:19. doi: 10.1186/s12942-019-0183-y (PMC6701131; doi:10.1186/s12942-019-0183-y)
Supplement: Supplementary file 1 — Additional file 1. Additional figures. [file 12942_2019_183_MOESM1_ESM.docx]

Supplement:

Figure S1: Nchelenge District sampled and enumerated households from April 2012 – July 2017. Taken with permission from Hast, M.A., et al. (2019). "The impact of three years of targeted IRS with pirimiphos-methyl on malaria parasite prevalence in a high-transmission area of northern Zambia." *The American Journal of Epidemiology.*


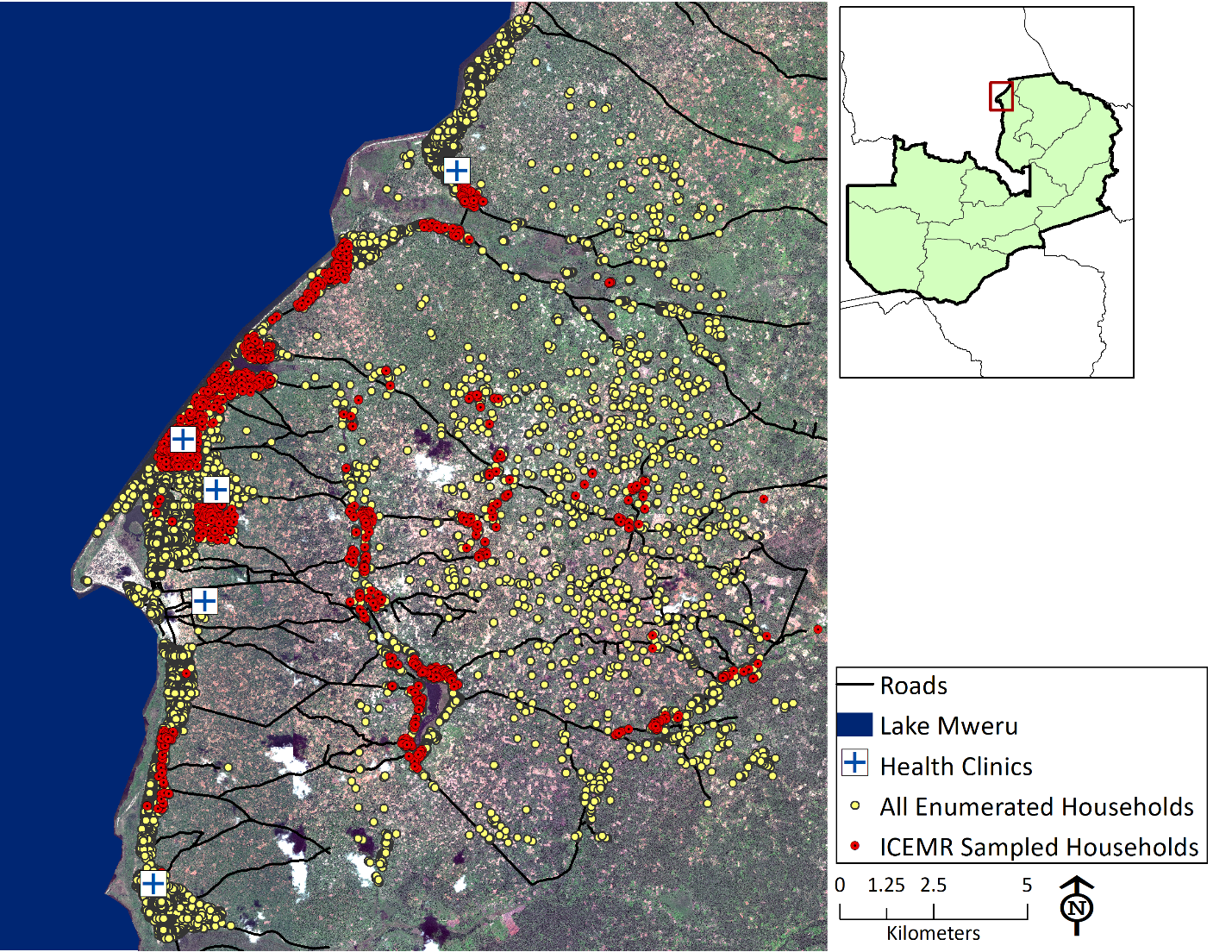


Figure S2: Histograms of total distance traveled in km and average daily distance traveled in km per participant, for overall time and peak biting time


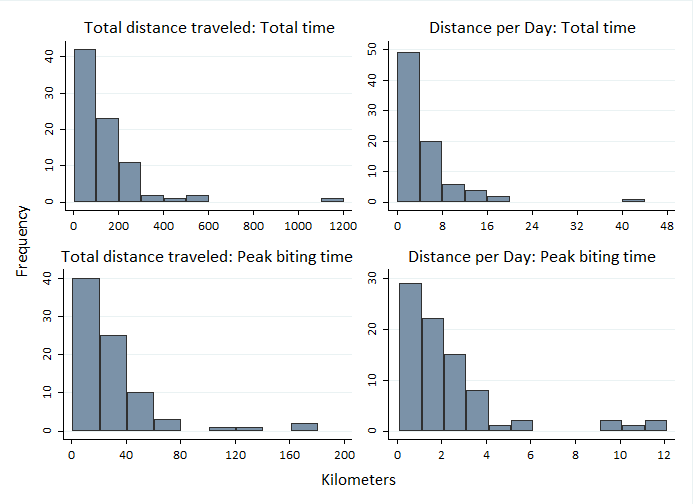


Figure S3: All GPS data logger points recorded from August 2014 to July 2015 in Nchelenge District overlaid on a malaria risk map of the study area


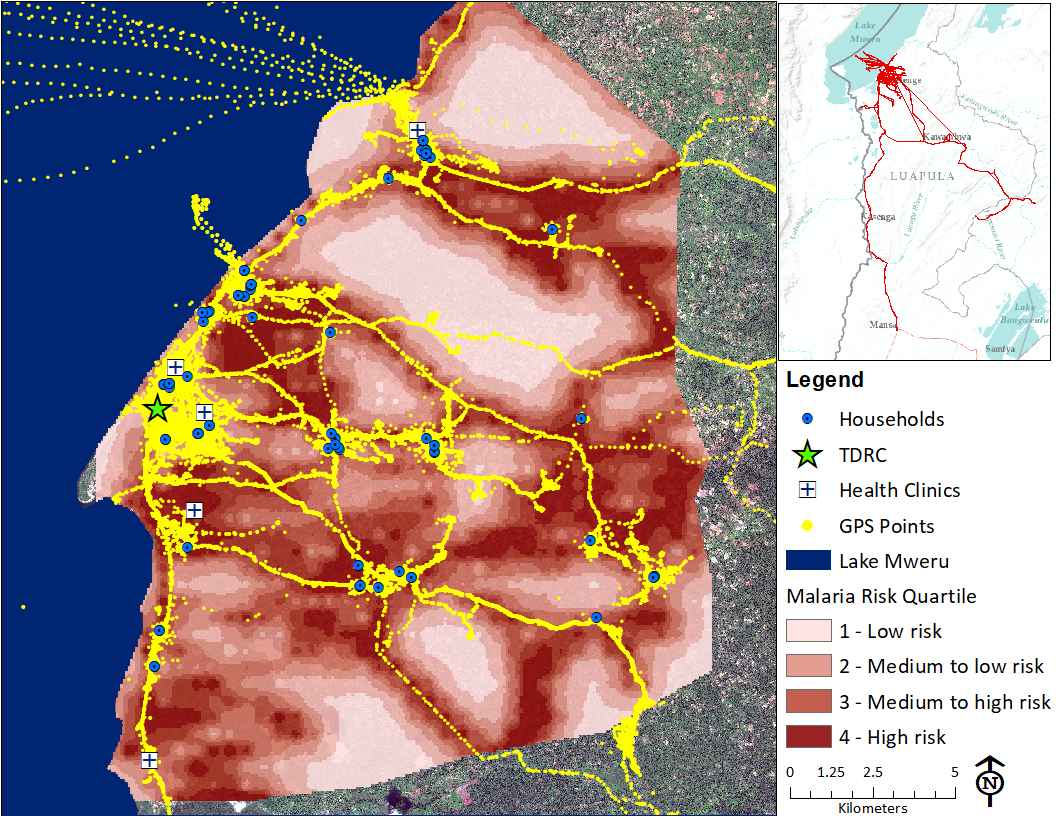


Figure S4: Boxplots of calculated average nightly malaria risk score by participant for A) peak biting times (*overall nightly risk*) and B) time spent away from home during peak biting times (*outdoor nightly risk*) by PCR status at visits 1 and 2

B

A
